# A general recognition theory model for identifying an ideal stimulus

**DOI:** 10.3758/s13414-022-02513-3

**Published:** 2022-06-14

**Authors:** Jeffrey B. Inglis, James Bird, F. Gregory Ashby

**Affiliations:** University of California, Santa Barbara, Santa Barbara, CA USA

**Keywords:** General recognition theory, Unfolding model, Ideal point, Preference, Concurrent ratings

## Abstract

A probabilistic, multidimensional model is described that accounts for sensory and hedonic ratings that are collected from the same experiment. The model combines a general recognition theory model of the sensory ratings with Coombs’ unfolding model of the hedonic ratings. The model uses sensory ratings to build a probabilistic, multidimensional representation of the sensory experiences elicited by exposure to each stimulus, and it also builds a similar representation of the hypothetical ideal stimulus in this same space. It accounts for hedonic ratings by measuring differences between the presented stimulus and the imagined ideal on each rated sensory dimension. Therefore, it provides precise estimates of the sensory qualities of the ideal on all rated sensory dimensions. The model is tested successfully against data from a new experiment.

## Introduction

Hedonic responses about a novel object are often based on the sensory characteristics of that object. Is the color pleasing? Does the curry have the right amount of heat? A popular model of such responses, called the unfolding model, was proposed more than 50 years ago by Coombs (1964). The unfolding model assumes that when judging one’s hedonic responses to a set of objects—for example, foods, beverages, or paintings—the observer imagines their ideal object within that category and then compares each object in the set to this imagined ideal. The objects are then ordered by preference according to their similarity to the ideal. So the most preferred object is the one that is most similar to the imagined ideal and the least preferred is the one that is least similar to the ideal. A wide variety of evidence supports this general model of preference (e.g., Chernev, 2003; Dubois, 1975; Rousseau, Ennis, & Rossi, 2012). The unfolding model has been generalized in a variety of different ways (e.g., Borg, 2018; DeSarbo & Rao, 1984; De Soete, Carroll, & DeSarbo, 1986; Ennis, 1993; Ennis & Johnson, 1994; Mullen & Ennis, 1991; Schönemann & Wang, 1972; Zinnes & Griggs, 1974), and applied successfully in a wide variety of different domains (e.g., Andrich, 1989; Davison, 1979; DeSarbo, Young, & Rangaswamy, 1997; Ennis & Rousseau, 2020; Roberts, Donoghue, & Laughlin, 2000).

The unfolding model provides an accurate account of preference orderings, but it is less successful at identifying the sensory characteristics associated with the imagined ideal. Some multidimensional versions of the model produce a multidimensional scaling (MDS) solution that situates each of the to-be-judged objects and the hypothetical ideal as a single point or probability distribution in a multidimensional space (e.g., De Soete et al.,, 1986; Zinnes & Griggs, 1974). However, as in traditional MDS, no information is provided about the nature of these dimensions. Sometimes, by noting which stimuli are situated at one extreme on a dimension and which stimuli are situated at the other extreme, it is possible to speculate about the nature of one or more dimensions. For example, if an MDS representation of odors places lemon and lavender at opposite ends of some dimension then one might infer that that dimension measures arousal (both are pleasant, but lemon is stimulating whereas lavender is calming). But with many dimensions, no such obvious ordering will emerge, and whatever inferences are made are generally impossible to test.

One experimental method for estimating the sensory characteristics of a stimulus, which is popular within the field of perception, is called the concurrent-ratings task. In this paradigm participants rate the magnitude of each stimulus simultaneously on a number of sensory dimensions, and then the observed ratings are used to estimate the participant’s sensory, perceptual, or cognitive impressions of the stimulus (Hirsch, Hylton, & Graham, 1982; Olzak, 1986). For example, consider an experiment in which participants first taste cups of coffee that were prepared using different amounts of ground coffee and different amounts of sugar. Next, the participants are asked to rate each cup on its sweetness and on the richness of its flavor (e.g., on a 1 to 7 scale). In this case the ratings would be used to estimate the sweetness and richness of each cup, and these representations could be used to judge whether sweetness interacts with richness, and to understand the psychophysical transformations from amount of sugar to perceived sweetness and amount of ground coffee to perceived richness.

When stimuli are rated on a single sensory dimension—most commonly sensory magnitude—the resulting data often can be modeled accurately by a signal-detection theory analysis. In fact, this is a popular experimental method for estimating an ROC curve (e.g., Ashby & Wenger, in press). When ratings are collected on multiple sensory dimensions, then the percepts are multivariate, rather than univariate, so the multidimensional generalization of signal-detection theory called general recognition theory (GRT; Ashby, 1988; Ashby & Townsend, 1986) is more appropriate. This analysis assumes that (1) the unobservable perceived values have a trial-by-trial (or participant-by-participant) multivariate normal distribution across the relevant sensory dimensions, (2) the participant establishes a set of criteria or cut-points on each rated dimension that partitions that dimension into intervals, and (3) a different numerical rating is assigned to each interval (Ashby, 1988; Wickens, 1992). This model assumes that on each trial, the participant determines in which interval the percept is in on each rated dimension and then selects the associated ratings.

Ashby and Ennis (2002) combined the unfolding model and the signal detection model of the ratings task to account for simultaneous sensory and liking ratings. This model used the participant’s sensory ratings to estimate the sensory representation of the ideal. However, the model was only developed and applied to situations in which the various stimuli all varied on a single sensory dimension. This article extends the model of Ashby and Ennis (2002) to more complex real-world stimuli that vary on many sensory dimensions. The resulting model estimates the distribution of imagined ideals (i.e., across trials and participants) by identifying the ideal mean on each rated sensory dimension and estimating the variance-covariance matrix of the ideal distribution across all rated dimensions.

The new model, which we call the GRT-unfolding model, is described in the next subsection. We then describe general methods for applying the model to data from an experiment that collects ratings on multiple sensory dimensions or attributes and on some hedonic dimension, such as liking. The methods and results sections describe an empirical test of the GRT-unfolding model against data from a new experiment. Finally, we discuss implications of our results and close with some brief conclusions.

### The GRT-unfolding model

**Fig. 1:**
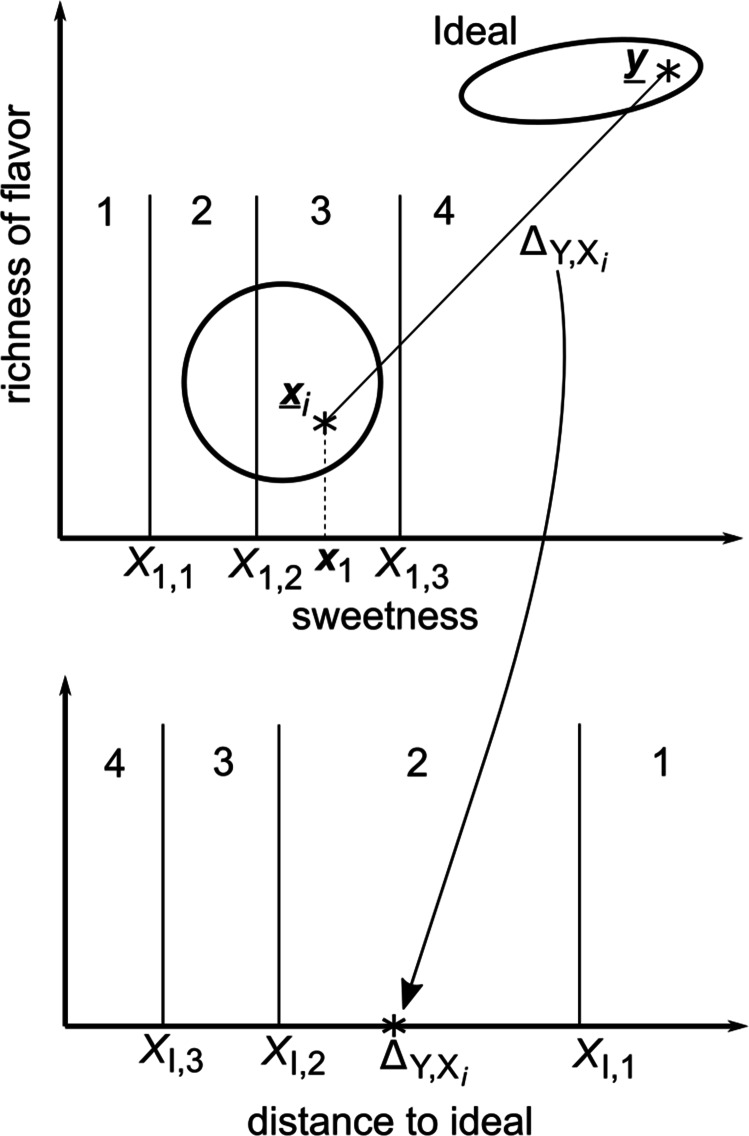
A schematic illustrating the GRT-unfolding model for a hypothetical trial in which a participant tastes a cup of coffee and then provides ratings (from 1 to 4) on the coffee’s sweetness and on liking. The circle and ellipse in the top panel are contours of equal likelihood from the sensory and ideal distributions, respectively. The participant’s responses on this trial are “3” on sweetness and “2” on liking

This section develops the GRT-unfolding model. An intuitive illustration of the assumptions underlying the model is provided in Fig. 1 for one hypothetical trial of a coffee-tasting experiment similar to the one described earlier. The only difference is that in this experiment participants are asked to rate: 1) the sweetness of the coffee; 2) the richness of the flavor; and 3) how much they like the coffee—all on 1 to 4 rating scales. The figure depicts hypothetical events on a trial in which the participant rates sweetness and liking, but not richness. The circle in the top panel is a contour of equal likelihood from the bivariate normal distribution that represents all possible percepts that are elicited by the specific cup of coffee that the participant tastes on this trial. It is a contour of equal likelihood because every point on this circle describes a percept that is equally likely to occur in any single tasting.^1^ The star labeled $\underline {\boldsymbol {x}}_{i}$ represents the specific percept experienced by the participant when tasting the current cup of coffee—that is, the specific perceived sweetness and richness of the current cup, which is the *i*^th^ cup of coffee in the experiment. Let ***x***_1_ denote the perceived sweetness and ***x***_2_ the richness of the flavor (i.e., so $\underline {\boldsymbol {x}}_{i} = [\boldsymbol {x}_{1}, \boldsymbol {x}_{2}]'$). The percept $\underline {\boldsymbol {x}}_{i}$ is assumed to be a random sample from the bivariate normal distribution that describes all possible percepts elicited by this cup. Note that the perceived sweetness of this particular cup (i.e., ***x***_1_) falls in the interval assigned to a rating of 3, so in this hypothetical example, the participant responds with a rating of 3 when asked to judge sweetness.

The model assumes that if the participant had been asked to rate the richness of the coffee’s flavor, rather than its sweetness, then the participant would have evaluated the position of the percept ***x***_2_ relative to the positions of three criteria established on the richness dimension (i.e., denoted *X*_2,1_,*X*_2,2_ and *X*_2,3_, respectively). These are not shown in Fig. 1 to keep the figure as simple as possible.

The tilted ellipse in the top panel of Fig. 1 is a contour of equal likelihood from the imagined ideal cup of coffee. Note that on average, the imagined ideal coffee is sweeter and has a richer flavor than the current cup. The star labeled $\underline {\boldsymbol {y}}$ denotes the sensory values of the imagined ideal on this trial, which again is assumed to be a random sample from the bivariate normal distribution that describes all possible imagined ideals. So note that the model predicts that because of a variety of different sources of variability (e.g., in preference and memory), the imagined ideal changes from trial to trial. The model assumes that to respond with a liking rating, the participant imagines the ideal cup of coffee, computes the distance (or similarity) of the current cup to this imagined ideal, and then responds with a rating based on this distance, with greater distances (or lower similarities) eliciting lower levels of liking and therefore smaller ratings. In Fig. 1, the distance falls in the interval assigned to a rating of 2 (see the bottom panel), so the participant responds with a liking rating of 2 on this trial.

More generally, consider an experiment in which participants are presented with *N* stimuli (one per trial) and each stimulus varies on *D* sensory dimensions. The goal is to collect ratings from 1 to *r* for each stimulus on the sensory strength for all *D* dimensions and on liking or some other hedonic response (with *r* representing maximum strength or maximum liking). In this general experiment, the GRT-unfolding model makes the following assumptions.

**Fig. 2:**
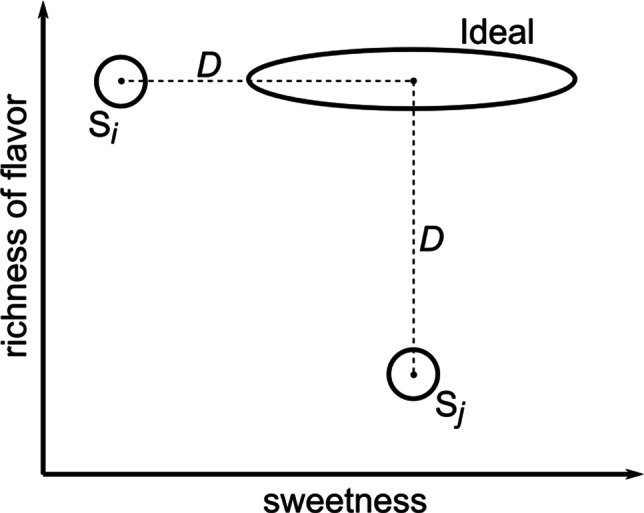
An example of how Mahalanobis distance weights the importance of psychological dimensions. Although the mean percepts elicited by cups of coffee S_*i*_ and S_*j*_ are the same Euclidean distance from the mean of the ideal cup, the S_*i*_ mean is closer according to Mahalanobis distance, and as a result, the GRT-unfolding model predicts that participants will usually like cup S_*i*_ more than cup S_*j*_

1. The sensory value on a trial when stimulus *i* is presented is represented by a *D* × 1 random vector $\underline {\boldsymbol {x}}_{i}$ in which $\underline {\boldsymbol {x}}_{i}^{\prime }=[\boldsymbol {x}_{1},\boldsymbol {x}_{2},...,\boldsymbol {x}_{D}]$, where ***x***_*d*_ represents the sensory magnitude on stimulus dimension *d*. Because of stimulus and perceptual noise and individual difference, $\underline {\boldsymbol {x}}_{i}$ varies randomly over trials and participants. We assume $\underline {\boldsymbol {x}}_{i}$ has a multivariate normal distribution with mean vector $\underline {\boldsymbol {\mu }}_{i}$ and variance-covariance matrix Σ_*i*_.
  Note that the variance-covariance matrix Σ_*i*_ contains *D*(*D* − 1)/2 covariances and *D* variances. For example, in the next section we consider an application of the GRT-unfolding model to an experiment in which participants rate the stimuli on 6 sensory dimensions. In this case, each Σ_*i*_ includes 15 covariances and 6 variances. If these are all free parameters then the model would include 27 parameters for each stimulus (15 covariances, 6 variances, and 6 means). These would require an enormous amount of data for accurate estimation. Furthermore, estimation of the covariances would require simultaneous ratings on all possible pairs of dimensions, plus the assumption that all of these ratings are based on the same sensory sample of the stimulus. Unfortunately, this assumption seems untenable. For example, if a participant is asked to rate a stimulus on 6 different dimensions then it seems likely that the participant would re-examine the stimulus one or more times before responding with all 6 ratings. According to the model, the sensory representation of the stimulus after each examination is represented by a new random sample $\underline {\boldsymbol {x}}_{i}$. If ratings on two dimensions are based on different $\underline {\boldsymbol {x}}_{i}$ samples then the correlation (e.g., across trials) between the ratings will not reflect the correlation between sensory dimensions.
  For these reasons, we only consider applications of the model to experimental paradigms in which a single one of the *D* + 1 ratings are requested on each trial, and each stimulus is presented to every participant on at least *D* + 1 different trials to ensure that all the necessary ratings are collected. In this case, no information about covariances is available, and as a result, we assume that all covariances equal 0 and therefore that Σ_*i*_ is diagonal. Furthermore, we also assume, without loss of generality, that all variances equal 1. This just serves to set the arbitrary unit of measurement on each dimension. Collectively, these assumption mean that, for all stimuli, Σ_*i*_ = I, where I is the identity matrix.
2. When asked to rate the sensory magnitude of the stimulus on dimension *d*, the participant constructs *r* − 1 response criteria, denoted *X*_*d*,1_,*X*_*d*,2_,...*X*_*d*,*r*− 1_, and responds with rating *j* if and only if *X*_*d*,*j*− 1_ < ***x***_*d*_ ≤ *X*_*d*,*j*_, where $X_{d,0}=-\infty $ and $X_{d,r}=\infty $. Note that in the Fig. 1 example, the perceived value of stimulus *i* on dimension 1 of this hypothetical trial (i.e., *x*_1_) lies between *X*_1,2_ and *X*_1,3_ and therefore the participant rates the sensory magnitude of this stimulus on dimension 1 as 3.
3. To generate a liking rating, the participant first imagines an ideal stimulus, which is represented by the *D* × 1 random vector $\underline {\boldsymbol {y}}$. Because of variability in the imagining process (e.g., due to variability in memory and affective state) and individual difference, $\underline {\boldsymbol {y}}$ varies randomly over trials and participants. We assume $\underline {\boldsymbol {y}}$ has a multivariate normal distribution with mean vector $\underline {\boldsymbol {\mu }}_{Y}$ and variance-covariance matrix Σ_*Y*_.
  The variance of the ideal distribution on each stimulus dimension is inversely related to the psychological importance of that dimension. If a dimension is important for liking, then the imagined ideal should have consistent values on that dimension. In contrast, if a dimension is unimportant for liking, then one would expect the imagined ideals to vary widely on that dimension. In the Fig. 1 example, note that the imagined ideal distribution has greater variance on sweetness than on richness, and that the values on these two dimensions have a slight positive correlation. The greater sweetness variance indicates that sweetness is less critical to liking than richness because when participants imagine their ideal cup of coffee they are more consistent in their imagined richness than in their imagined ideal level of sweetness.
4. The participant computes the Mahalanobis distance ${\Delta }_{Y,X_{i}}$ between the imagined ideal $\underline {\boldsymbol {y}}$ and the sensory value $\underline {\boldsymbol {x}}_{i}$ (from step 1). As we will see, this is just regular Euclidean distance except each dimension is weighted by its psychological importance to the ideal, where importance is measured by the inverse of the variance of the ideal distribution on that dimension (as described in point 3).
  Figure 2 shows an example of this dimensional weighting by importance. Note that in this example, the ideal distribution has smaller variance on the richness dimension than on sweetness, indicating that participants treat richness as a more important criterion of their imagined ideal cup of coffee than sweetness. The two circles denote contours of equal likelihood from the perceptual distributions of two actual cups of coffee—S_*i*_ and S_*j*_. Note that the means of these distributions are both the same Euclidean distance from the mean of the ideal distribution (i.e., a distance of *D*). Even so, the units of Euclidean distance are the same as the units of the sensory space, whereas the units of Mahalanobis distance are standard deviations of the ideal distribution. Therefore, percepts elicited by cup S_*i*_ will tend to be closer according to Mahalanobis distance to imagined ideal cups of coffee than percepts elicited by cup S_*j*_ because the S_*i*_ percepts are fewer standard deviations from the ideal than the S_*j*_ percepts (i.e., the ideal standard deviation is larger on the sweetness dimension than on richness). As a result, the GRT-unfolding model predicts that participants will usually like cup S_*i*_ more than cup S_*j*_, despite the fact that both cups are the same Euclidean distance from the ideal mean.
5. The participant constructs *r* − 1 response criteria, denoted *X*_I,1_,*X*_I,2_,...*X*_I,*r*− 1_, and responds with liking rating *j* if and only if $X_{\text {I},j} < {\Delta }_{Y,X_{i}} \leq X_{\text {I},j-1}$, where $X_{I,0}=\infty $ and *X*_*I*,*r*_ = 0. Note that in the Fig. 1 example, the distance between the imagined ideal and the perceived stimulus (i.e., ${\Delta }_{Y,X_{i}}$) lies between *X*_I,2_ and *X*_I,1_ and therefore the participant responds with a liking rating of 2.

### Fitting the model to data

For each stimulus, the data can be collected as a (*D* + 1) × *r* matrix in which the entry in row *d* and column *j* is the frequency that participants assigned rating *j* to the stimulus on dimension *d*, where row *D* + 1 is liking. Note that each matrix has (*D* + 1) × (*r* − 1) degrees of freedom, since there is one constraint per row (i.e., each row sum equals the number of trials that participants rated the stimulus on the attribute associated with that row). There is one such matrix for each of the *N* stimuli, so overall, the data include *N* × (*D* + 1) × (*r* − 1) degrees of freedom.

The model predicts that the probability that rating *j* is assigned to stimulus *i* on sensory dimension *d* equals the area under the dimension *d* marginal pdf of $\underline {\boldsymbol {x}}_{i}$ between *X*_*d*,*j*− 1_ and *X*_*d*,*j*_. Because these marginal distributions are all normal, each of these probabilities can be computed via straightforward z transformations and appeal to the cumulative z distribution function.

Computing the predicted probabilities of various liking ratings is considerably more difficult. The predicted probability that participants assign stimulus *i* a liking rating of *j* equals

1 $$ P_{\text{L}}(j | S_{i}) = P(X_{\text{I},j} < {\Delta}_{Y,X_{i}} \leq X_{\text{I},j-1}), $$

where, as before, ${\Delta }_{Y,X_{i}}$ is the Mahalanobis distance between the imagined ideal $\underline {\boldsymbol {y}}$ and the sensory value $\underline {\boldsymbol {x}}_{i}$. Since ${\Delta }_{Y,X_{i}}$ is nonnegative, note that

2 $$ \begin{array}{@{}rcl@{}} P_{\text{L}}(j | S_{i}) &= &P(X_{\text{I},j} < {\Delta}_{Y,X_{i}} \leq X_{\text{I},j-1}) \\ &=& P(X_{\text{I},j}^{2} < {\Delta}_{Y,X_{i}}^{2} \leq X_{\text{I},j-1}^{2}). \end{array} $$

Now

3 $$ \begin{array}{@{}rcl@{}} {\Delta}_{Y,X_{i}}^{2} &=& (\underline{\boldsymbol{y}} - \underline{\boldsymbol{x}}_{i})' {\Sigma}_{Y}^{-1} (\underline{\boldsymbol{y}} - \underline{\boldsymbol{x}}_{i}) \\ &=& \underline{\boldsymbol{w}}' {\Sigma}_{Y}^{-1} \underline{\boldsymbol{w}}, \end{array} $$

where $\underline {\boldsymbol {w}} = \underline {\boldsymbol {y}} - \underline {\boldsymbol {x}}_{i}$ is a multivariate normally distributed random vector with mean vector $\underline {\boldsymbol {\mu }}_{W} = \underline {\boldsymbol {\mu }}_{Y} - \underline {\boldsymbol {\mu }}_{i}$ and variance-covariance matrix Σ_*W*_ = Σ_*Y*_ + I.

The random variable ${\Delta }_{Y,X_{i}}^{2}$ defined by Eq. 3 has the distribution of a weighted sum of *D* non-central *χ*^2^ random variables, each with one degree of freedom (Scheffé, 1999). Efficient numerical integration algorithms are available to compute the Eq. 2 probability under these distributional assumptions (e.g., de Micheaux & de Micheaux, 2017). However, *D* = 6 in the application described in the next section, which is large enough so that this weighted sum could be considered approximately normally distributed. Therefore, to implement the normal approximation to the Eq. 2 probability, we need only to compute the mean and variance of ${\Delta }_{Y,X_{i}}^{2}$.

The Appendix shows that the Eq. 3 random variable has mean

4 $$ \mu_{{\Delta}^{2}} =D + \text{trace}({\Sigma}_{Y}^{-1}) + (\underline{\boldsymbol{\mu}}_{Y} - \underline{\boldsymbol{\mu}}_{i})' {\Sigma}_{Y}^{-1} (\underline{\boldsymbol{\mu}}_{Y} - \underline{\boldsymbol{\mu}}_{i})  $$

and variance

5 $$ \begin{array}{@{}rcl@{}} \sigma_{{\Delta}^{2}}^{2} &=&2D + 4\text{trace}({\Sigma}_{Y}^{-1}) + 2\text{trace}({\Sigma}_{Y}^{-2}) \\ &&+ 4 (\underline{\boldsymbol{\mu}}_{Y} - \underline{\boldsymbol{\mu}}_{i})' {\Sigma}_{Y}^{-1}(\text{I} + {\Sigma}_{Y}^{-1}) (\underline{\boldsymbol{\mu}}_{Y} - \underline{\boldsymbol{\mu}}_{i}). \end{array} $$

Therefore, we can approximate the predicted probability that rating *j* is assigned to stimulus *i* on the liking dimension by computing the area between $X_{\text {I},j}^{2}$ and $X_{\text {I},j-1}^{2}$ under the pdf of a normal distribution with mean and variance specified by Eqs. 4 and 5, respectively.

### An empirical application

**Fig. 3:**
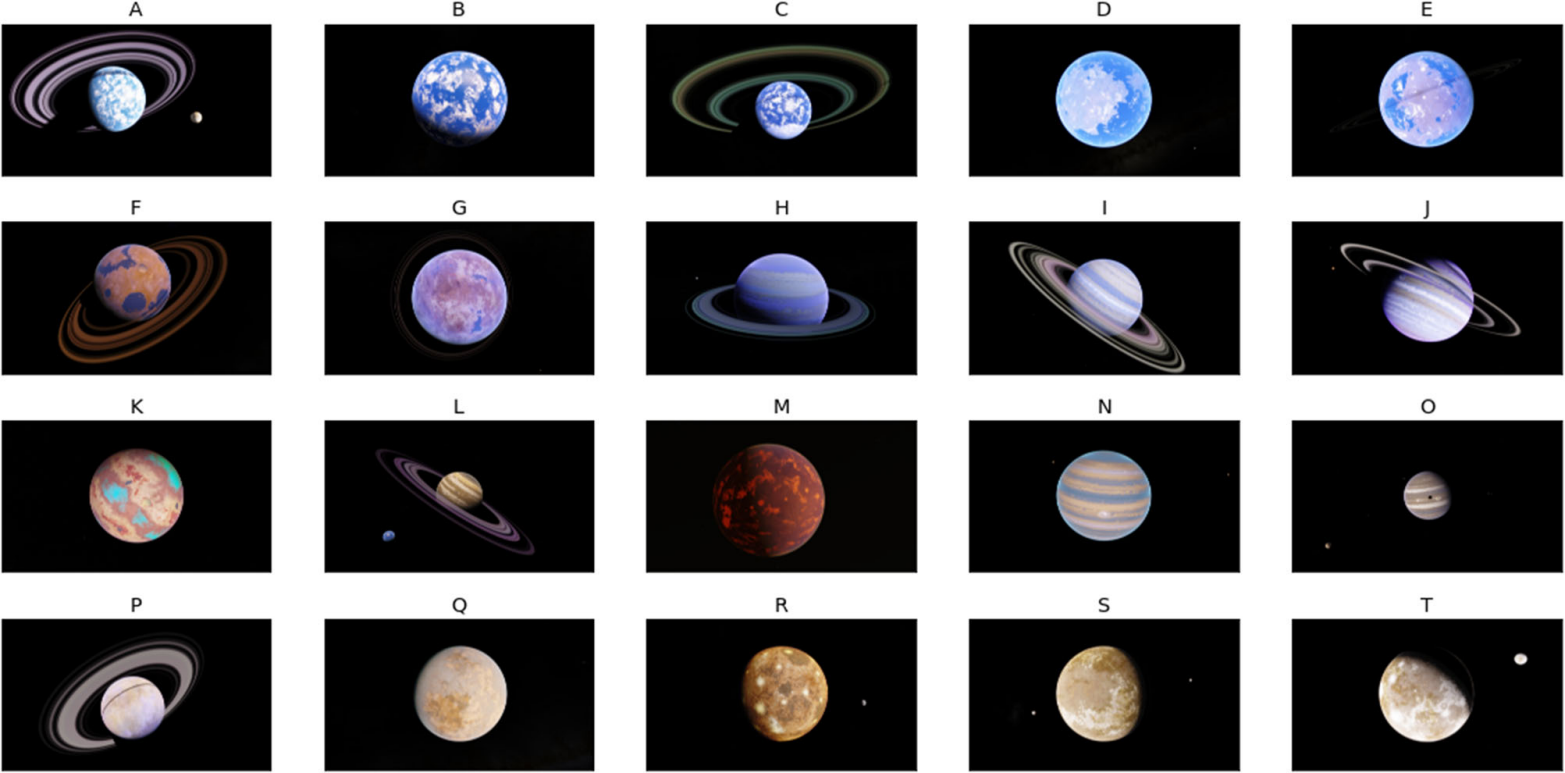
The 20 planets shown to each participant

As an empirical test of the model, we ran an experiment in which 29 participants rated the 20 images of hypothetical planets shown in Fig. 3 on six sensory dimensions and on an hedonic dimension. Specifically, participants were told to imagine that they were in a spaceship traveling through deep space, and that their mission was to rate planets they encountered (from 1 to 7) on the prominence of a number of sensory dimensions (water, clouds, rings, moons, blue-green, red-yellow) and on how important it was to retain a photograph of the planet and send it back to earth.

## Method

### Stimuli

All images were gathered using SpaceEngine (SpaceEngine.org), a universe simulator that randomly generates a plethora of astronomical objects. The procedural generation process creates 3-dimensional rendered planets, which are captured with extreme detail using a 3840 × 21604*K* resolution and resulting in over 8 million pixels per image. Due to the stochastic nature of each image, the options for planetary features and combinations are nearly limitless. The stimuli used in this experiment are displayed in Fig. 3.

### Participants

Twenty-nine students at the University of California, Santa Barbara participated in an (approximately) one-hour experiment in exchange for course credit. All participants had normal color vision and were sampled randomly from a population in which 62% self-reported as female, 42% as a racial minority, and 21% as Hispanic or Latino. All relevant ethical regulations were followed and the study protocol was approved by the Human Subjects Committee at UCSB. Informed consent was obtained from all participants, and every participant was allowed to quit the experiment at any time for any reason and still receive credit.

### Procedure

Participants were told to imagine that they were in a spaceship traveling through deep space and that the ship automatically takes photos of planets that it encounters. They were also told that their mission was to rate each planet on a number of physical attributes and on how important they thought it was to send the image back to earth so that the rest of humanity would know of that planet’s existence. Participants were presented the images in 5 phases. During each phase, the 20 images were displayed one-at-a-time in a random order. In phase 1, participants passively observed the images. In phases 2–5, each image was displayed with a ratings bar that ranged from 1 to 7 and participants were instructed to move the mouse and click the integer on the ratings bar that agreed with their rating. During phases 2 and 4, the image and ratings bar were accompanied by a word cue that specified the physical attribute to be rated, such as “water”. Participants rated 6 different attributes (or dimensions) and each attribute/image combination was presented once per phase, resulting in a total of 240 sensory judgments during phases 2 and 4 (2 sensory judgments per planet per dimension). During phases 3 and 5, the image and ratings bar were accompanied by the word cue “importance”. Prior to each phase, participants were reminded that their job was to use the mouse to click the value on the scale that best reflected the prominence of the feature indicated by the word cue, with 1 being least prominent and 7 being most prominent. Each image was presented once during phases 3 and 5, resulting in 2 importance judgments per planet.

A few participants were not sufficiently engaged in some phases of the experiment. These participants tended to repeat the same rating, over and over. Therefore, any importance phase (3 and 5) in which the participant emitted 3 or fewer unique ratings was excluded from analysis. Six liking phases were excluded, leaving 52 liking phases for analysis. Additionally, any sensory phase (2 and 4) in which the participant gave the same rating on any dimension to all images was excluded from analysis. Two sensory phases were excluded, resulting in 56 sensory phases for analysis.

Any model of these data could only recover psychologically meaningful representations if the sample sizes are large enough to provide accurate estimation of the true rating probabilities. The frequency with which participants assign rating *j* to stimulus *S*_*i*_ has a multinomial distribution with variance *Np*(1 − *p*), where *N* is the number of ratings collected on stimulus *S*_*i*_ and *p* is the true probability that stimulus *S*_*i*_ is rated *j*. Therefore, the standard error of the proportion that estimates *p* is $\sqrt {p(1-p)/N}$. With 7-point rating scales and a heterogeneous sample of participants, the true rating probabilities would all equal *p* = 1/7 = .014. Under this null hypothesis, a sample size of *N* = 48 results in a standard error of approximately 0.05. Our smallest sample size was larger than this (i.e., *N* = 52), hence under this same null hypothesis, our standard errors of measurement would all be less than 0.05.

## Results

**Fig. 4:**
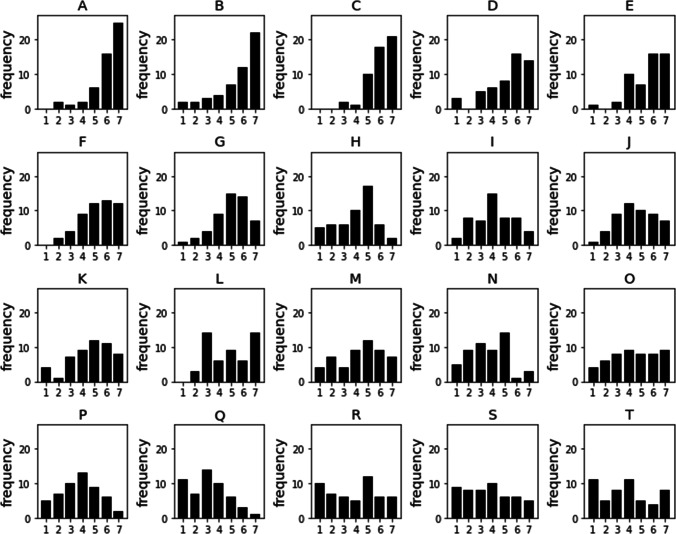
Importance ratings for each of the 20 planets

The data from this experiment were aggregated across participants and then recorded in a 20 (planets) × 7 (dimensions) × 7 (ratings) frequency array, where importance was included as one of the 7 dimensions. The importance ratings for each planet are shown in Fig. 4. For each planet and dimension, the frequency sum across the 7 ratings equals the number of trials participants were asked to rate that planet on that dimension. Therefore, the data include 6 degrees of freedom for each planet and dimension, and so the entire data set includes 840 degrees of freedom (i.e., 20 × 7 × 6).

The GRT-unfolding model was fit to these data. The model included a total of 183 free parameters. Without loss of generality, we fixed the mean vector for planet N, $\underline {\boldsymbol {\mu }}_{N}$, to the zero vector. In addition, as described earlier, to limit the number of free parameters we fixed the variance-covariance matrices of all sensory distributions to Σ_*i*_ = I. The following parameters were all free to vary:

1. The remaining 19 mean vectors, $\underline {\boldsymbol {\mu }}_{i}$ for all *i*≠*N*. Each $\underline {\boldsymbol {\mu }}_{i}$ is 6 × 1, so there were a total of 114 free mean parameters (i.e., 19 × 6).
2. Six criteria, *X*_*d*,*j*_, on each of the 6 sensory dimensions, resulting in an additional 36 parameters.
3. Six means for the ideal distribution, $\underline {\boldsymbol {\mu }}_{Y}$,
4. The 6 × 6 ideal variance-covariance matrix, Σ_*Y*_ (21 free parameters).
5. Six criteria, *X*_I,*j*_, on the squared-distance-to-ideal dimension.

All parameters were estimated via constrained optimization by linear approximation (COBYLA; Powell, 1994) using SciPy (Virtanen et al., 2020) in Python (Van Rossum & Drake, 1995) by minimizing the sum of squared errors between the predicted and observed response frequencies.

Overall, the GRT-unfolding model accounted for 95.27% of the variance in the data (*r*^2^=.9527). Although the model included 183 free parameters, because the data had 840 degrees of freedom, after parameter estimation, there were still 657 degrees of freedom left to test the model (i.e., 840 − 183). So accounting for 95% of the variance in these 657 proportions seems impressive. Not surprisingly, however, the model was more successful at accounting for the sensory ratings than the liking ratings. Specifically, the GRT-unfolding model accounted for 96.09% of the variance in the sensory ratings data and 73.67% of the variance in the liking ratings.

**Fig. 5:**
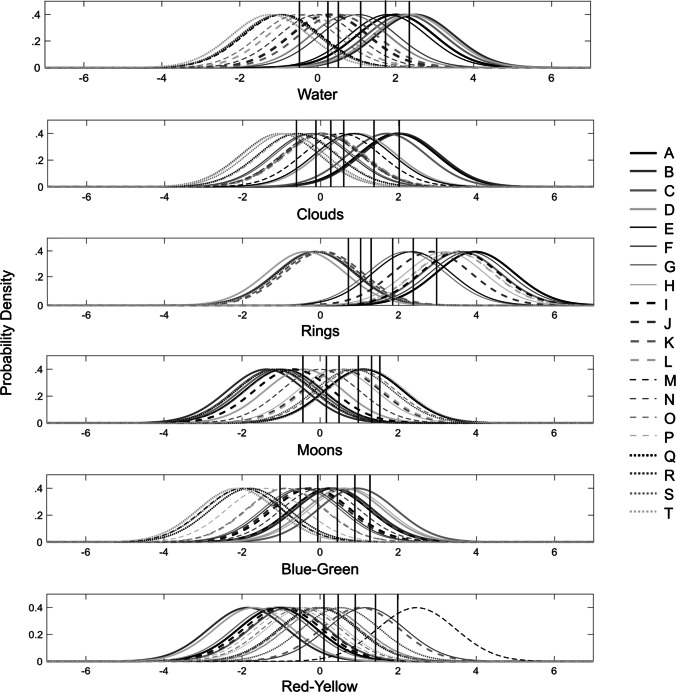
Estimated sensory distributions from the best-fitting version of the GRT-unfolding model, along with the estimated criteria on each dimension that participants used to assign ratings

Figure 5 shows estimated sensory distributions for each planet on each dimension as well as the estimated criteria. Note that, except for rings, the planets vary fairly continuously on all sensory dimensions. Not surprisingly, the perceived prominence of rings is approximately bimodal with some planets displaying prominent rings (e.g., planets A, C, and F) and other planets showing a prominent absence of rings (e.g., planets B, D, and S).

Table 1 shows the variance-covariance matrix of the estimated ideal distribution. The variances provide an inverse measure of how important each dimension is to the ideal. Note that the smallest variance is on the clouds dimension and the next smallest is on water. The small variances suggest that when ideal planets are imagined on different trials, participants always tend to imagine a planet with similar values on the water and cloud dimensions. In contrast, the variances on the red-yellow and moons dimensions are large, suggesting that the different imagined ideals vary widely on the red-yellow and moons dimensions. Therefore, for example, if the imagined ideal sometimes has a moon and sometimes does not, then the presence or absence of a moon is not an important attribute of the ideal planet.

**Table 1:** Variance-covariance matrix of the ideal distribution from the best-fitting version of the GRT-unfolding model

	Water	Clouds	Rings	Moons	Blue-Green	Red-Yellow
Water	33.65	− 20.46	33.27	29.46	30.45	− 10.09
Clouds		23.09	− 23.32	− 10.77	1.46	1.78
Rings			191.14	245.11	83.73	21.00
Moons				558.85	113.26	66.89
Blue-Green					145.61	20.25
Red-Yellow						214.29

**Fig. 6:**
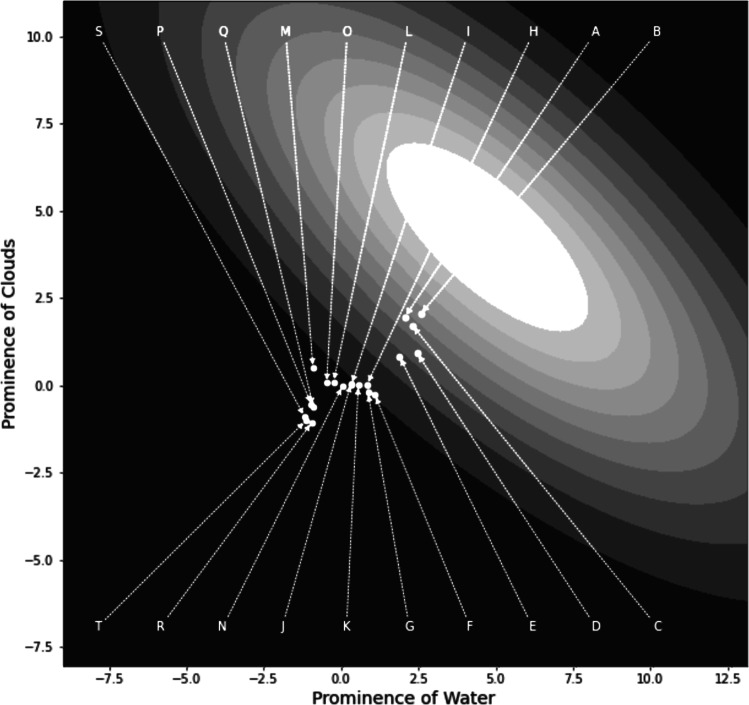
Contours of equal likelihood of the ideal distribution from the best-fitting version of the GRT-unfolding model (i.e., the ellipses) and the sensory means of each planet projected onto the plane defined by the water and cloud dimensions

Figure 6 shows the ideal distribution and the mean of each planet distribution projected onto the plane defined by the two most important sensory dimensions—namely, water and clouds. The ellipses denote the contours of equal likelihood of the ideal distribution, so the ideal mean lies at the center of these ellipses. Note from Table 1 that water and clouds are negatively correlated in the ideal distribution, which is the reason that the ellipses in Fig. 6 have a negative orientation. This makes sense because as cloud cover increases there is less available surface to display water. Note that planet B is closest to the ideal mean, closely followed by planets A and C, and that planets R, S, and T are furthest (i.e., see Fig. 3 for ordering relative to the ideal when considering all dimensions). Therefore, these data suggest that the ideal planet would have a greater prominence of cloud cover and water than any of the planets that were shown to participants.

## Discussion

The GRT-unfolding model uses sensory ratings to build a probabilistic, multidimensional representation of the sensory experiences elicited by exposure to each stimulus. If participants rate the stimuli on *D* sensory dimensions then the sensory representations built by the model will be *D* dimensional. And the model will also build a representation of the ideal stimulus in this same space. It then attempts to account for hedonic ratings by measuring differences between the presented stimulus and the imagined ideal on each of these *D* sensory dimensions. This approach can only hope to account for the hedonic responses of participants if the rated sensory dimensions include all stimulus attributes that significantly affect the hedonic response. To take an extreme example, consider an experiment in which participants rate a set of stimuli on *D* sensory dimensions but that the participants’ hedonic responses to those stimuli depend exclusively on some other, unrated sensory dimension. In this case, the hedonic responses will be independent of the stimulus value on any of the rated dimensions, and therefore a comparison of the stimulus to the ideal values on the *D* rated dimensions will not predict the participant’s hedonic response. So the efficacy of the GRT-unfolding model depends strongly on the ability of the experimenter to identify all sensory dimensions that could significantly affect the hedonic responses of participants to the selected stimuli.

Given this, the default expectation should be that the model will account for sensory ratings better than it accounts for hedonic ratings. In the experiment described here, the GRT-unfolding model accounted for 96% of the variance in the sensory ratings and 74% of the variance in the hedonic ratings. Therefore, we believe that one plausible account for this difference is that participants based their hedonic responses, at least in part, on some unrated dimension or attribute of the planets. Traditional multidimensional unfolding models that lack any sensory data could just add more unspecified dimensions to the model until goodness-of-fit is maximized (e.g., exactly as in MDS). Even so, note that a better fit by such a model would provide only vague information about the sensory qualities of the ideal. Given that the GRT-unfolding model provides precise estimates of the sensory qualities of the ideal on all rated sensory dimensions, we believe that accounting for 74% of the variance in the hedonic ratings is impressive, especially since the model was provided no information about how participants might make these judgments.

Another limitation of the model is that it implicitly assumes that all of the rated sensory dimensions are separable, rather than integral. The classic definition is that two stimulus dimensions are separable if it is possible to attend to one and ignore variation in the other (Garner, 1974; Shepard, 1964). If this is impossible then the dimensions are integral. Classic separable dimensions are color and shape. The judgments that observers make about the color of an object are unaffected if the shape of the object changes from circle to square. Classic integral dimensions are the brightness and saturation of a color patch. Brightness judgments change when the saturation of the color patch changes. In ratings experiments, participants are asked to rate the presented stimulus on single sensory dimensions. This requires them to allocate all attention to that dimension and ignore all other sensory values of the stimulus. The classic account is that such selective attention is possible only if the rated dimension is separable from the other sensory dimensions.

GRT distinguishes between perceptual and decisional separability. Perceptual separability holds if the perception of a stimulus component is unaffected by changes in the level of some other component, whereas decisional separability holds if the decision about the level of a component is unaffected by changes in the perceived value of the other component (Ashby & Townsend, 1986). Technically, the GRT-unfolding model assumes that decisional separability holds, but it makes no assumptions about perceptual separability. In the Fig. 1 example, the vertical decision bounds that the model assumes participants use to assign sensory ratings satisfy decisional separability because, for example, the criterion used to decide whether the sweetness of the sample should be rated as “1” versus “2” does not depend on the richness of the sample. Participants can learn to use strategies that satisfy decisional separability, even with the prototypically integral dimensions of brightness and saturation (Ell, Ashby, & Hutchinson, 2012). As a result, assuming decisional separability is considerably weaker than assuming perceptual separability. Even so, with integral stimulus dimensions, more variability should be expected in the decision strategies participants use to provide ratings on the various sensory dimensions, which could reduce the overall efficacy of the model.

As described earlier, a number of multivariate generalizations of the unfolding model have been proposed (De Soete et al., 1986; Ennis, 1993; Ennis & Johnson, 1994; Mullen & Ennis, 1991; Zinnes & Griggs, 1974). Despite the theoretical value of these models, Ennis and Ennis (2013) suggested three reasons why these generalizations have not had a greater practical impact. First, the models require pairwise comparisons that can be expensive to obtain (e.g., “which do you prefer, A or B”). The GRT-unfolding model avoids this criticism because it only requires hedonic and sensory ratings on single stimuli. For example, with the 20 planets used in our experiment, pairwise comparisons would require collecting ratings on 190 different pairs (i.e., (20 × 19)/2), whereas the GRT-unfolding model only requires ratings on the 20 individual planets. Therefore, the GRT-unfolding model requires far fewer trials than previous models, and the data it does require readily can be collected remotely via any of several widely available current software packages.

On the other hand, it is relatively straightforward to extend the GRT-unfolding model to paired-comparisons data. The key is to use some intermediary model that converts paired-comparisons proportions to single-stimulus scale values. A simple and obvious option for this step is the Luce (1959) choice model. For example, suppose the proportion of participants who prefer stimulus A to stimulus B equals *P*(*A*,*B*). Then according to the Luce (1959) choice model, there exist non-negative numerical values *v*_*A*_ and *v*_*B*_ for which

6 $$ P(A,B)=\frac{v_{A}}{v_{A} + v_{B}}.  $$

Since the *v*_*i*_ lie on a ratio scale, the unit of measurement is arbitrary. As a result, without loss of generality, one of the *v*_*i*_ can be set to 1. Given this constraint, it is straightforward to estimate a *v*_*i*_ for each stimulus from the paired-comparisons data.

Ratings data are categorical since the responses of observers are restricted to some finite set of integer values (e.g., some integer from 1 to 7). The response criteria shown in Fig. 1 provide a model of how these categorical responses are generated from the underlying continuous perceptual representations. In contrast, Eq. 6 provides continuous measures of the judged dimension. In the case of preference judgments, the GRT-unfolding model would interpret these values as continuous measures of the similarity of each stimulus to the ideal. For example, according to the similarity model proposed by Ashby and Perrin (1988), the preference score *v*_*i*_ would be interpreted as the amount of overlap between the ideal and stimulus *i* perceptual distributions. The final step in generalizing the GRT-unfolding model to paired-comparisons data would be to estimate the parameters of the ideal distribution from the set of distributional overlaps associated with all of the stimuli used in the paired-comparisons experiment. Another advantage of this generalization is that many preference judgments are context dependent (e.g., Tversky & Simonson, 1993), and the Ashby and Perrin (1988) similarity model provides a mechanism to model such dependencies.

Ennis and Ennis (2013) noted that a second reason that previous models have not had a greater practical impact is that they are mathematically complex, which makes them difficult to apply. In contrast, the GRT-unfolding model is simple to apply due to the normal approximation to the squared Mahalanobis distance between the sensory representation of the stimulus and the imagined ideal. This approximation reduces the complexity of the model significantly since the probability of the various sensory and liking ratings can be computed via straightforward z transformations and appeal to the cumulative z distribution function. Hopefully, these advantages will lead to more applications of the GRT-unfolding model to academic and industry data sets.

Third, Ennis and Ennis (2013) noted that another reason that previous multidimensional unfolding models are not more popular is because they do not generate individual-level ideal representations. This is largely due to the enormous amount of data they require (e.g., because they rely on paired-comparisons experiments). As we just noted, the GRT-unfolding model requires much less data and therefore is much less susceptible to this problem. Nevertheless, because the GRT-unfolding model requires ratings on each identified sensory dimension, the amount of data it requires increases (linearly) with the number of rated sensory dimensions. Therefore, whereas individual ideal representations should be straightforward to estimate in applications where only a few sensory dimensions require ratings, estimating individual ideal representations is more problematic when many sensory dimensions are required. For example, in our empirical application to planets, we collected ratings on 6 sensory dimensions, which was too many to allow the model to be fit to individual participant data when each participant completed only a single 50-minute experimental session.

If individual ideal representations are desired, then there are several options. One, of course, is to collect sufficient data from each participant to allow the model to be fit to individual-participant data—either by increasing the length of the experimental session or increasing the number of sessions. A second option is to reduce the number of rated sensory dimensions, which would increase the number of ratings that could be collected on each dimension in a single session. The trick here is to eliminate dimensions that do not affect the participant’s hedonic response. One approach might be to run an initial group experiment with many dimensions, fit the model to the group data, and then use these results to identify the key sensory dimensions. For example, the variances listed in Table 1 indicate that in our experiment, Moons had little or no effect on hedonic ratings, and Rings and Red-Yellow had at most a minimal effect. Therefore, a follow-up experiment that asked for ratings only on the Water, Clouds, and Blue-Green sensory dimensions might be able to collect enough data to allow estimation of individual ideals at the cost of only a minimal decrease in goodness-of-fit. Another approach to reducing the number of sensory dimensions is to consult someone with expertise with the stimuli (e.g., a Master Sommelier in the case of wines).

Finally, a third option is to estimate an ideal representation, not for individual participants, but for groups of similar participants, or perhaps for groups of similar stimuli. For example, the ideal may change if the coffee is made with some flavored coffee bean. In either case, a separate experiment is required for each identified group, but each participant in these experiments only needs to complete a single experimental session. This approach seems especially relevant for product design, since industries do not create unique products for each individual, but they might create a product that is tailor-made for one particular segment of consumers.

As an empirical test of the GRT-unfolding model, we chose the planets shown in Fig. 3 because they are interesting, real-world objects. However, the GRT-unfolding model could be applied to any stimuli. Because the model estimates the sensory values of the ideal stimulus, it has the potential to greatly benefit product development. In many cases, the sensation elicited by a stimulus on an identified sensory dimension is directly related to some underlying physical quantity. For example, the sweetness of a Merlot wine is related to its residual sugar content (among other factors). Therefore, identifying the ideal sweetness of a Merlot could facilitate the efforts of vintners to create more popular wines.

## Appendix

The mean of the Eq. 3 random variable is (e.g., Khatri, 1980; Paolella, 2018)

7 $$ \begin{array}{@{}rcl@{}} \mu_{{\Delta}^{2}} &=& \text{trace} ({\Sigma}_{Y}^{-1}{\Sigma}_{\underline{\text{w}}}) + \underline{\boldsymbol{\mu}}_{\underline{\text{w}}}^{\prime} {\Sigma}_{Y}^{-1} \underline{\boldsymbol{\mu}}_{\underline{\text{w}}} \\ &=& \text{trace}[{\Sigma}_{Y}^{-1}({\Sigma}_{Y} + \text{I})] + (\underline{\boldsymbol{\mu}}_{Y} - \underline{\boldsymbol{\mu}}_{i})^{\prime} {\Sigma}_{Y}^{-1} (\underline{\boldsymbol{\mu}}_{Y} - \underline{\boldsymbol{\mu}}_{i}) \\ &=&D + \text{trace}({\Sigma}_{Y}^{-1}) + (\underline{\boldsymbol{\mu}}_{Y} - \underline{\boldsymbol{\mu}}_{i})^{\prime} {\Sigma}_{Y}^{-1} (\underline{\boldsymbol{\mu}}_{Y} - \underline{\boldsymbol{\mu}}_{i}). \end{array} $$

Furthermore, if we let *λ*_*i*_ denote the *i*^th^ eigenvalue of Σ_*Y*_, then Eq. 7 reduces to

8 $$ \mu_{{\Delta}^{2}} = D + \sum\limits_{i=1}^{D} \lambda_{i}^{-1} + (\underline{\boldsymbol{\mu}}_{Y} - \underline{\boldsymbol{\mu}}_{i})^{\prime} {\Sigma}_{Y}^{-1} (\underline{\boldsymbol{\mu}}_{Y} - \underline{\boldsymbol{\mu}}_{i}).  $$

Note that the last term is just the squared Mahalanobis distance between the means of the two distributions.

The variance of the Eq. 3 random variable is (e.g., Khatri, 1980; Paolella, 2018)

9 $$ \begin{array}{@{}rcl@{}} \sigma_{{\Delta}^{2}}^{2} &=& 2 \text{trace} [({\Sigma}_{Y}^{-1}{\Sigma}_{W})^{2}] + 4 \underline{\boldsymbol{\mu}}_{W}^{\prime} {\Sigma}_{Y}^{-1} {\Sigma}_{W} {\Sigma}_{Y}^{-1} \underline{\boldsymbol{\mu}}_{W} \\ &=& 2 \text{trace}[{\Sigma}_{Y}^{-1}({\Sigma}_{Y} + \text{I}){\Sigma}_{Y}^{-1} ({\Sigma}_{Y} + \text{I})]\\ &&+ 4 (\underline{\boldsymbol{\mu}}_{Y} - \underline{\boldsymbol{\mu}}_{i})^{\prime} {\Sigma}_{Y}^{-1} ({\Sigma}_{Y} + \text{I}) {\Sigma}_{Y}^{-1} (\underline{\boldsymbol{\mu}}_{Y} - \underline{\boldsymbol{\mu}}_{i}) \\ &=&2 \text{trace}(\text{I} + 2 {\Sigma}_{Y}^{-1} + {\Sigma}_{Y}^{-2}) + 4 (\underline{\boldsymbol{\mu}}_{Y} - \underline{\boldsymbol{\mu}}_{i})^{\prime} {\Sigma}_{Y}^{-1}\\ &&(\text{I} + {\Sigma}_{Y}^{-1}) (\underline{\boldsymbol{\mu}}_{Y} - \underline{\boldsymbol{\mu}}_{i}) \\ &=&2D + 4\text{trace}({\Sigma}_{Y}^{-1}) + 2\text{trace}({\Sigma}_{Y}^{-2}) + 4 (\underline{\boldsymbol{\mu}}_{Y} - \underline{\boldsymbol{\mu}}_{i})^{\prime} {\Sigma}_{Y}^{-1}\\ &&(\text{I} + {\Sigma}_{Y}^{-1}) (\underline{\boldsymbol{\mu}}_{Y} - \underline{\boldsymbol{\mu}}_{i}). \end{array} $$

If expressed in terms of the eigenvalues of Σ_*Y*_, then Eq. 9 becomes

10 $$ \begin{array}{@{}rcl@{}} \sigma_{{\Delta}^{2}}^{2} &=& 2D + 4\sum\limits_{i=1}^{D} \lambda_{i}^{-1} + 2\sum\limits_{i=1}^{D} \lambda_{i}^{-2} + 4 (\underline{\boldsymbol{\mu}}_{Y} - \underline{\boldsymbol{\mu}}_{i})^{\prime} {\Sigma}_{Y}^{-1}\\ &&(\text{I} + {\Sigma}_{Y}^{-1}) (\underline{\boldsymbol{\mu}}_{Y} - \underline{\boldsymbol{\mu}}_{i}) \\ &=& 2D + 2\sum\limits_{i=1}^{D} (2\lambda_{i}^{-1} + \lambda_{i}^{-2}) + 4 (\underline{\boldsymbol{\mu}}_{Y} - \underline{\boldsymbol{\mu}}_{i})^{\prime} {\Sigma}_{Y}^{-1}\\ &&(\text{I} + {\Sigma}_{Y}^{-1}) (\underline{\boldsymbol{\mu}}_{Y} - \underline{\boldsymbol{\mu}}_{i}). \end{array} $$

Therefore, we can approximate the predicted probability that rating *j* is assigned to stimulus *i* on the liking dimension by computing the area between $X_{\text {I},j}^{2}$ and $X_{\text {I},j-1}^{2}$ under the pdf of a normal distribution with mean and variance specified by Eqs. 7 and 9, respectively (or alternatively, by Eqs. 8 and 10, respectively).

## Footnotes

1 Points inside the circle are more likely to occur than points on the circle, and points outside the circle are less likely to occur.
